# A Test of Canine Olfactory Capacity: Comparing Various Dog Breeds and Wolves in a Natural Detection Task

**DOI:** 10.1371/journal.pone.0154087

**Published:** 2016-05-06

**Authors:** Zita Polgár, Mari Kinnunen, Dóra Újváry, Ádám Miklósi, Márta Gácsi

**Affiliations:** 1 Department of Ethology, Eötvös Loránd University, Budapest, Hungary; 2 Department of Ecology, University of Oulu, Oulu, Finland; 3 Institute for Wildlife Conservation, Faculty of Agriculture and Environmental Sciences, Szent István University, Gödöllő, Hungary; 4 MTA-ELTE Comparative Ethology Research Group, Budapest, Hungary; GI Lab, UNITED STATES

## Abstract

Many dog breeds are bred specifically for increased performance in scent-based tasks. Whether dogs bred for this purpose have higher olfactory capacities than other dogs, or even wolves with whom they share a common ancestor, has not yet been studied. Indeed, there is no standard test for assessing canine olfactory ability. This study aimed to create a simple procedure that requires no pre-training and to use it to measure differences in olfactory capacity across four groups of canines: (1) dog breeds that have been selected for their scenting ability; (2) dog breeds that have been bred for other purposes; (3) dog breeds with exaggerated short-nosed features; and (4) hand-reared grey wolves. The procedure involved baiting a container with raw turkey meat and placing it under one of four identical ceramic pots. Subjects were led along the row of pots and were tasked with determining by olfaction alone which of them contained the bait. There were five levels of increasing difficulty determined by the number of holes on the container’s lid. A subsample of both dogs and wolves was retested to assess reliability. The results showed that breeds selected for scent work were better than both short-nosed and non-scent breeds. In the most difficult level, wolves and scenting breeds performed better than chance, while non-scenting and short-nosed breeds did not. In the retested samples wolves improved their success; however, dogs showed no change in their performances indicating that a single test may be reliable enough to assess their capacity. Overall, we revealed measurable differences between dog breeds in their olfactory abilities and suggest that the Natural Detection Task is a good foundation for developing an efficient way of quantifying them.

## Introduction

Wolves rely heavily on their sense of smell to function in the world around them, using it to identify each other, mark their territories, and to locate prey [1–3]. As both wolves and our modern-day canine companions evolved from a common ancestor, we can presume that they share many of these inherent abilities to use olfaction for a variety of tasks. However, how the processes of domestication and selective breeding has altered the dogs’ utilization of these abilities, and indeed the abilities themselves, is unclear and worthy of investigation (see e.g. [4]).

Domestic dogs are used in a wide variety of scent detection tasks with great success in explosives and drug detection in the military and police forces [5,6], cancer detection in the medical field [7,8], trailing humans for search and rescue [9], and tracking animals for hunting and conservation [10,11]. Dogs have been reported to be able to discriminate between the odors of twins who differed either in environmental factors (mainly diet) or in genetic relatedness (fraternal twins), however, they were unable to discriminate between odors produced by infant twins identical in both genetic relatedness and environmental factors [12].

Certain breeds have been bred to excel in such olfaction-based tasks, and while the acuity with which they are able to perform them is impressive, it is unclear how much better they are when compared to other dogs who have not been bred for such purposes, as there have been no systematic comparative studies. In a study to evaluate the reliability of bloodhounds in identifying and trailing the scent of individual humans in high-traffic areas on 48-hour-old trails, five trained and experienced bloodhounds had a success rate of 96% with no false identifications [13]. Although the bloodhound may be well suited for the variation of scent discrimination used in trailing, this ability is not limited to this breed. Labrador retrievers, German shepherd dogs and Belgian shepherds are also used in scenting work because of their drive and high trainability.

Many of the breeds selected for their scenting abilities, particularly scent-hounds, have been shown to be more difficult to train than certain other breeds [14,15]. Examining their olfactory abilities could provide a greater understanding into whether the extended training of these breeds for use in certain tasks is truly warranted, or if other easier to train breeds could perform the olfactory element of those tasks just as well.

Using handler questionnaires as well as behavior studies assessing accuracy scores of detection dogs has provided mixed results about breed differences in olfactory performance. Handlers seem to be equally satisfied with four of the most common types of dogs used for search tasks (English springer spaniel, Labrador retriever, Border collie, and cross breeds), however this is based on overall performance, including characteristics like obedience, stamina, intelligence and playfulness, rather than just accuracy in scent detection [16]. Meanwhile, an efficacy study on four breeds of drug detection dogs (German shepherd, Labrador retriever, “terriers”, English cocker spaniel) found that German shepherds had higher accuracy than the other three breeds, with the terriers performing least accurately [5].

So far, no thorough investigation of the genetic background of different dog breeds’ olfactory abilities has been carried out. Although earlier studies have not found differences in the number of olfactory receptor genes between scent-hounds, sight-hounds, and toy breeds [17], more recent studies have reported that differences do exist in the polymorphism of those genes [18,19]. While these studies lack behavioral data to back up their findings—making it impossible to determine whether the differences manifest at the phenotypic level as well—one study did find that police dogs (primarily German shepherd dogs) with a specific substitution at one gene location were rated as having significantly better scent detection skills than other dogs who had had the same training but did not have this substitution [20]. This provides some support for the idea that genetic differences may affect scenting ability even within breeds on the individual level.

Currently, there is no standard test for assessing canine olfactory capacity. Previous studies that have tried to measure this ability have generally relied on training a small sample of dogs to indicate the presence of a biologically irrelevant scent among other irrelevant scents on some form of testing platform or device [21–25]. One example is using a "line-up" system where the dog is faced with a match-to-sample task where they must indicate which option in a line-up contains the target scent [8]. While these studies provide an important starting point for olfactory research, they are not practical for a number of reasons. For example, as the scents are irrelevant to the dogs, the tasks require extensive training. This makes the tests not only time consuming, but also brings into question whether it is truly olfaction that is being tested and not trainability, as any results obtained are not only dependent on the individual dog’s olfactory abilities but also on their cognitive skills, motivation, memory, and attention span. It also means that there is no way of assessing a dog’s abilities before investing the time to train them, which is important when selecting potential working dogs. Indeed, in some programs as many as half of the dogs or more chosen for training in scent tasks fail to meet certification criteria [25,26]. While some of these failures are due to temperament or training issues, many are due to inadequate performance on scenting tests, indicating that a simple relative olfactory assessment that would allow trainers to choose the best individuals from a group prior to the start of training would be of great practical use.

In light of this, the aims of our study were to (1) create a practical Natural Detection Task that could be used for assessing the olfactory abilities of a wide variety of untrained canines, and in turn to (2) quantify relative differences in olfactory sensitivity through controlled testing with odorants of decreasing thresholds. By doing this, we enable the comparison of the relative detection thresholds of different breed groups and even hand raised wolves. The main question we sought to answer was whether there are quantifiable differences between the olfactory abilities of scent breeds (dogs bred specifically for the purpose of performing scent-related jobs), non-scent breeds (dog breeds which have been selected for jobs that require characteristics not related to olfaction) and short-nosed breeds (dog breeds that have been selected to have drastically reduced nasal features). To answer this question we developed a simple procedure, the Natural Detection Task, where the animals had to find a standard amount of food—raw meat—in a natural searching/choice situation that was gradually made more difficult with each level. In addition we sought to compare the detection thresholds of these different breed groups to a population of captive hand raised and extensively socialized grey wolves. As these wolves had been born into captivity and thus have not had the need to rely on their olfactory abilities to hunt or survive, this allowed us to assume that any differences in ability are likely due to genetic and/or morphological differences. We assumed that wolves would perform better than dogs due to the relaxed selection related to olfactory abilities in dogs, but not necessarily better than the scent group, because later directional selection for scenting abilities might have counterbalanced the effects of domestication. To check whether the Natural Detection Task had consistently measured the subjects’ olfactory capacity we retested a subsample of both dogs and wolves.

## Methods

### Ethics statement

This research was conducted based on the written approval (PEI/001/1058-4/2015) of the Pest County Governmental Office, Hungary. All owners and animals took part in our studies voluntarily and were free to stop participating at any time. Owners signed consent forms where they indicated that they understood the test procedures and agreed to allow their dogs and/or wolves to take part in the study.

### Subjects

Out of the 13 hand raised and extensively socialized grey wolves (for detailed rearing conditions see [27]) and 49 dogs that were motivated by the bait, one wolf and seven dogs did not finish all trials of the test (5 x 4 trials). Thus the subjects analyzed in this study were 12 wolves (8 males, 4 females; average age 8 ± 1 years), and 41 adult pet dogs. The dogs were divided in to three groups.

Scent dogs: The first group consisted of 14 individuals from breeds selected specifically for scent work (basset hound (3), beagle (3), German pointer (3), wire-haired vizsla (2), bracco Italiano, grand basset griffon Vendéen, Transylvanian hound; 9 males, 5 females; mean age 5.7 ± 0.8 years),

Non-scent dogs: The second group consisted of 15 individuals from breeds not selected for scent work (bichon Havanese (2), Chinese crested powder puff (2), English greyhound (2), Hungarian greyhound (2), whippet (2), Afghan hound, bichon Bolognese, greyhound cross, miniature pincher, Siberian husky; 9 males, 6 females; mean age 5 ± 1 years).

Short-nosed dogs: The third group consisted of 12 individuals from short-nosed breeds (Cavalier King Charles spaniel (4), Boston terrier (2), boxer (2), American bulldog/boxer cross, bullmastiff, English bulldog, pug; 7 males, 5 females; mean age 4.8 ± 0.7 years).

The dogs were recruited on a volunteer basis from the Family Dog Project database and the Top Mancs Dog School, Budapest. The tested dogs were regularly fed raw meat by their owners, so the bait was not a novel food to them (raw meat was used in this study as it was the only bait that was motivating for the wolves). All the dogs were companion dogs living with families, although two animals in the non-scent group were in training to be hearing dogs for the deaf. An additional three dogs in the scent group as well as one dog in the short-nosed group had received some basic training in man-trailing, but were not certified for Search and Rescue purposes. The wolves were from the Horkai Animal Training Center, Gödöllő, where they were kept in small packs and occasionally used for filming purposes. Wolves were not fed on the day before the test was carried out, dog owners were asked not to feed their dogs in the twelve hours before the test. An additional eight animals (one wolf and seven dogs—three in scent group, two in non-scent group, two in short-nosed group) were excluded because they did not complete the entire test (these are not included in the above lists—details can be found in the S1 Dataset).

To assess whether experience in the test situation would increase the subjects’ performance, we retested a subsample of five wolves and seven dogs (one in the scent group, four in the non-scent group, and two in the short-nosed group) at least a week after their original tests. This semi random sample of subjects was chosen only from those individuals who scored less than 100% in Level 5 (see later).

### Location and weather

As the wolves could not be brought indoors, the tests were run outdoors for all subjects in flat grassy areas that were familiar to the animals. These areas were surrounded by hedges, making them relatively visually isolated from potential distractions. They were also areas that were not frequented by dog-walkers, minimizing the amount of distracting scents. The tests were run during the months of May and June 2014 on days with similar weather with an average temperature of 23 ± 1 degrees Celsius and an average humidity of 47 ± 2%. There was no significant difference between the average weather conditions between the groups (one-way ANOVA—temperature: F_(3, 46)_ = 1.9, p = 0.143; humidity: F_(3,44)_ = 0.99, p = 0.406).

### Procedure

**Fig 1:**
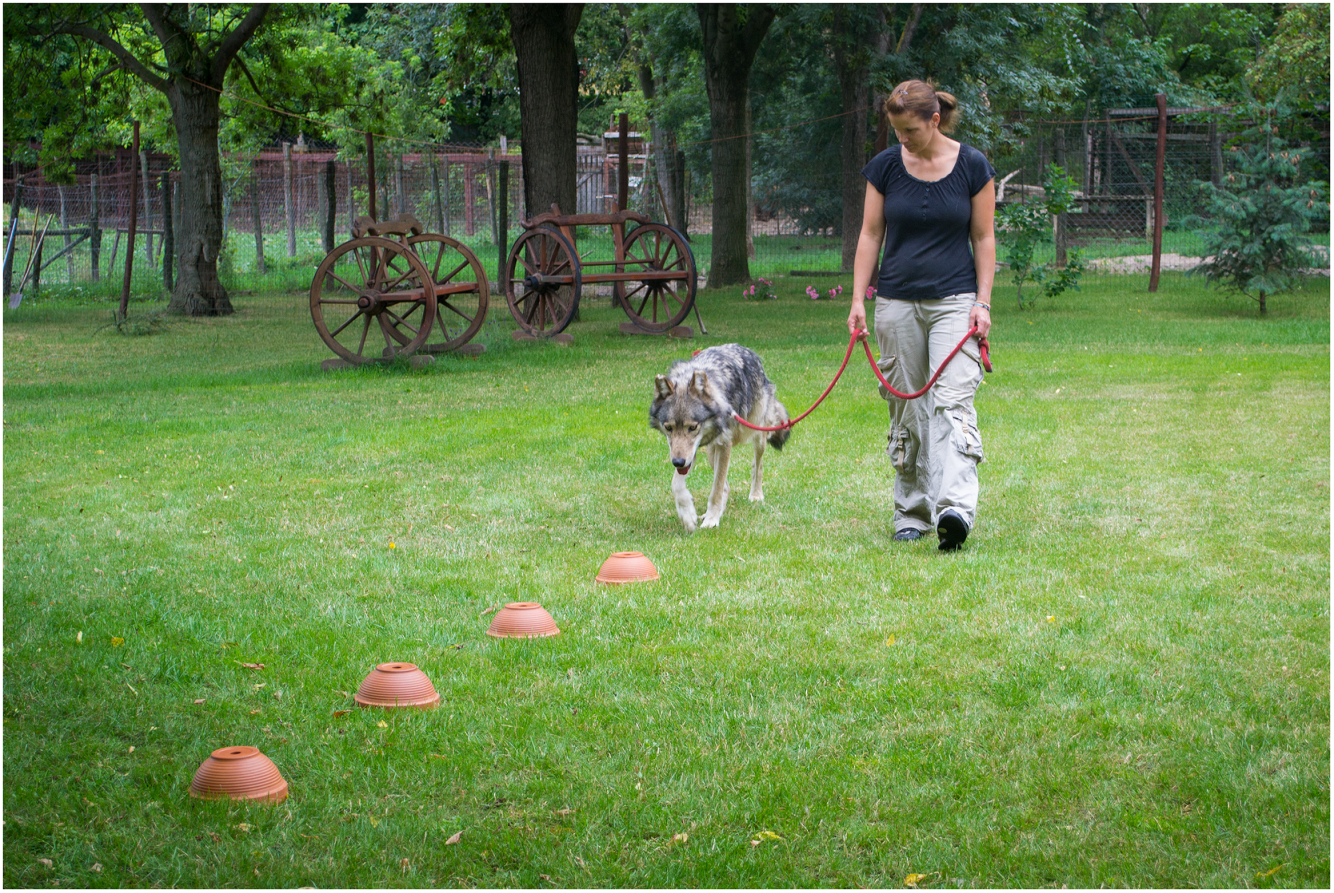
The test setup. One of the containers placed under the four upside down ceramic pots, placed at approximately one-meter intervals, is baited with 30–32 grams of raw meat. The owner is leading a wolf along the pots.

Four round ceramic pots (21 cm diameter) each with a 2.5 cm hole on the bottom were placed upside-down in a straight line at approximately one-meter intervals. Under each pot was a round plastic container (Curver brand, 0.5 l) with a lid. The test situation was set up as shown in Fig 1.

Three of the containers were marked as “no food” containers and never came into contact with any food or any persons that handled the food. A number of other containers were marked as “food” and were used for baiting.

**Fig 2:**
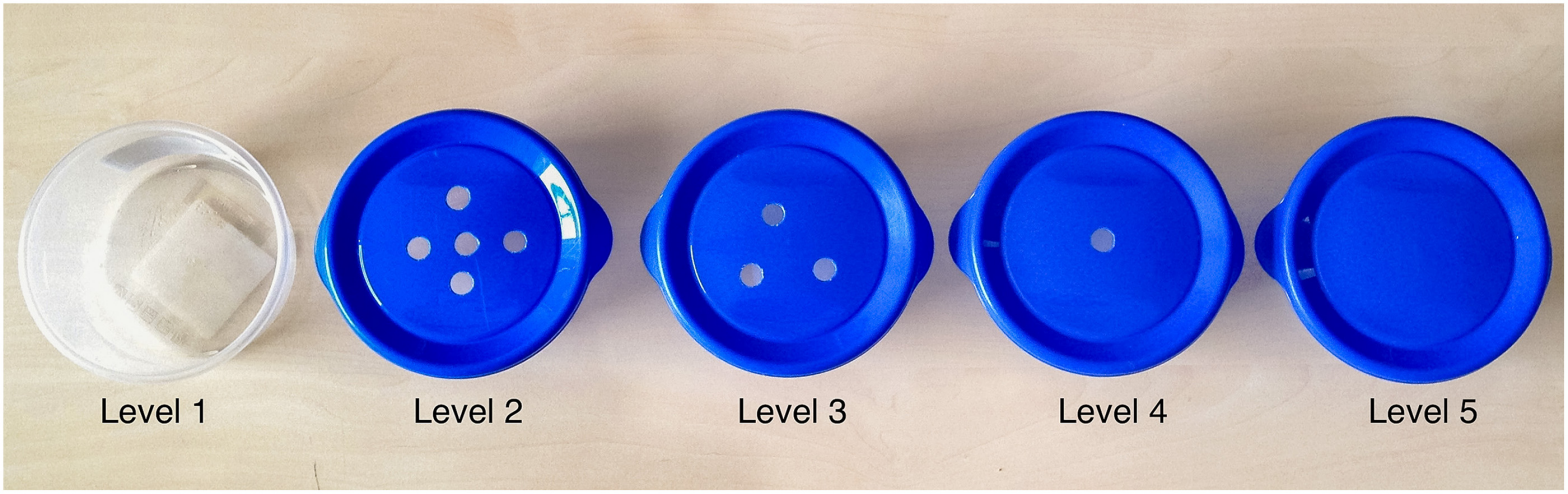
Levels of difficulty. The different types of lids used for baiting in the five levels of difficulty. Each hole was 1 cm in diameter.

There were five levels of difficulty that were determined by what type of lid was placed on the baited container (Fig 2). In Level 1, the container had an open top with no lid. In Levels 2–4 the lids had five, three, or one 1 cm holes cut into them. In Level 5, the container was covered completely with a regular lid (no holes). There were four trials for each level (a total of 20 trials), where the baited container was under each of the four pots once in a random order. The bait was never under the same pot twice consecutively. Based on experiences from the pilot studies and in order to facilitate rapid learning of the task, a step-down procedure of increasingly difficult levels was chosen in favor of a randomized order of difficulty.

Once the line of pots had been set up, a measuring cup was used to place the raw turkey leg meat (30-32g kept at temperatures between 3–5°C, purchased always from the same stock, included only flesh and no bone) into the designated container, which was then covered with the appropriate pot. A clean container was used for every trial. The surface area of the meat in the container was approximately 5cm^2^.

Two experimenters were needed to run the test. E1 set up the pots and placed the baited container in the predetermined position. She never came into contact with the meat. E2 handled the meat and never came into contact with the pots or the “no food” containers. She was also blind to the location of the baited container. The camera was held either by a third person or by E1. The owner and the animal did not view the baiting of the pots and faced away from the test area until they were called.

As soon as the meat was placed, a stopwatch was started. After one minute had elapsed, E1 called the owner and the animal to come to the starting line and they were allowed to begin walking the path (on loose leash). Before they started walking, the owner was allowed to give an encouraging command (such as “search!”, “sniff!”, or “go find!”), however she was not permitted to say anything or make any gestures once they began walking or while the animal was examining the pots. The only exception was if the animal walked away from the test setup, in which case the owner could call the animal back by saying “come here, where’s the food?” in an encouraging tone. Furthermore, if at the beginning of the test the animal showed no interest in examining the pots, E2 gave the dog a small piece of meat and then, while the dog was watching, walked the line of pots and pretended to manipulate each of them, but did not actually touch any of them.

While walking along the setup, the animal could smell each of the pots. The trial was ended either when the animal indicated one of the pots or one minute of active searching had elapsed without indication. An indication was defined as any of the following behaviors: placing paw on pot; refusing to move away from pot; attempting to turn pot over; vocalizing while next to pot; significantly increasing tail wagging speed while sniffing pot; or sitting next to the pot (see S1 and S2 Videos for examples). An indication was only counted if it lasted more than 2s. If the animal began to move on from a pot after an indication lasting less than 2s, it was allowed to keep searching. If the owner and the animal reached the end of the line without making an indication, and the minute had not yet elapsed, they were permitted to turn back around and walk the line from the opposite direction until the time was up. The presence of an indication was determined by E2, who was blind to the location of the food.

The results of each trial could be classified as being in one of four categories. The choice was *correct* if the animal indicated the baited pot within the allowed time. The choice could be incorrect by being either *false*, if the animal indicated an unbaited pot, or a *no choice*, if the animal examined all four pots but did not make an indication within one minute of active searching. The trial was recorded as *no attempt* if the animal did not examine or show interest in the pots or if the animal repeatedly moved away from the row. If there were three consecutive *no attempts*, the test was ended and the subject was excluded from the study.

If the animal indicated correctly, it was allowed to eat the meat from the container. E1 removed the pot and the owner opened the container and allowed the animal to eat the meat. Both the touched ceramic pot and container were replaced with clean ones for the next trial. If the animal indicated incorrectly (*false choice*), then the owner showed him that the indicated pot contained no food. If the animal did not choose (*no choice* or *no attempt*) then the animal was not shown any of the pots.

In order to decrease the likelihood that the animals would use cognitive strategies or local enhancement to indicate at a pot, after each trial the row of pots was rotated to a different angle and shifted over at least 1m by E1 on the field. The starting line was also shifted to the opposite side for each trial.

### Data collection & statistical analysis

A handheld camera recorded the trials. The results of each trial were classified and noted on the spot and controlled from the video records later. The success rate at each level (number correct out of four) for each individual was calculated.

We analyzed the effect of two factors: the *group* (scent dog, non-scent dog, short nosed dog, and wolf) and the *level* of the test (1–5) as main effects, and also their two-way interaction on the success (average score at each level) by generalized linear mixed models (GLMM) with binomial distribution and logit link. The performance of the different groups at Level 5 was compared to chance through one-sample Wilcoxon signed rank test.

We compared the test-retest results using a GLMM for the overall success of the two groups (wolf and mixed dog sample) in the whole test. Then—using another GLMM—we further analyzed the wolves’ test-retest data comparing their success by levels.

## Results

**Fig 3:**
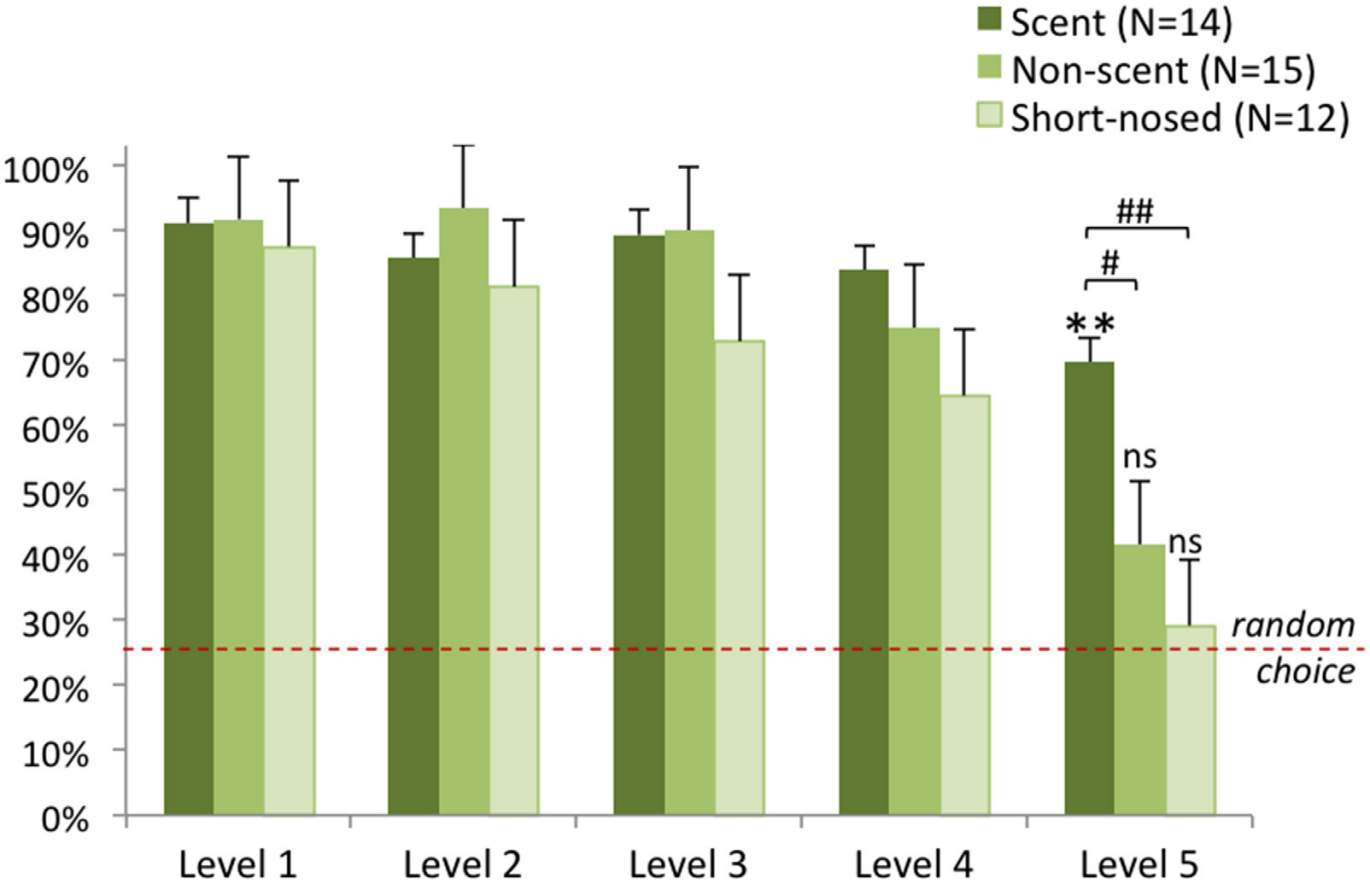
Performance of dogs in the Natural Detection Task. Mean (SE) performances for the three dog groups across the five levels are shown. Dashed line represents chance level; Level 5 difference from chance: ** P<0.01. Level 5 difference between groups: # P = 0.053; ## P<0.01.

The generalized linear mixed model showed significant effect: F_(19:245)_ = 6.724, p<0.001. We found significant interaction between the effect of the level and the group (F_(12:245)_ = 1.975, p = 0.027). The Sidak post hoc tests revealed significant difference only at Level 5; the performance of the scent dog group was better than that of the short-nosed dog group (t_(246)_ = 3.621, p = 0.002) and the non-scent dog group (t_(246)_ = 2.569, p = 0.053) (Fig 3).

While at Level 5 the performance of non-scent and short nosed dogs did not differ from chance (non-scent: p = 0.145; short nosed: p = 0.564), scent dogs and wolves were better than chance (scent: p = 0.004; wolf: p = 0.018).

The breakdown of the percentages of incorrect choices that were either *false* or *no choice* for each group at each level is shown in Table 1. Only the excluded animals had *no attempt* choices so these are not included. Due to the differing contribution of the number of subjects vs. number of trials per group with *no choice* responses, these values have not been compared statistically.

**Table 1:** Percentages of incorrect choices that were ‘false’ and ‘no choice’ for each group at each level.

Group	Wolf (n = 12)		Scent (n = 14)		Non-Scent (n = 15)		Short Nosed (n = 12)	
	False Choice	No Choice	False Choice	No Choice	False Choice	No Choice	False Choice	No Choice
**Level 1**	15%	0%	9%	2%	2%	6%	8%	0%
**Level 2**	15%	0%	16%	2%	6%	0%	11%	0%
**Level 3**	15%	0%	13%	0%	9%	0%	15%	1%
**Level 4**	24%	0%	20%	0%	13%	11%	20%	1%
**Level 5**	29%	0%	33%	4%	23%	30%	35%	8%
**Total**	100%	0%	91%	9%	53%	47%	90%	10%

**Fig 4:**
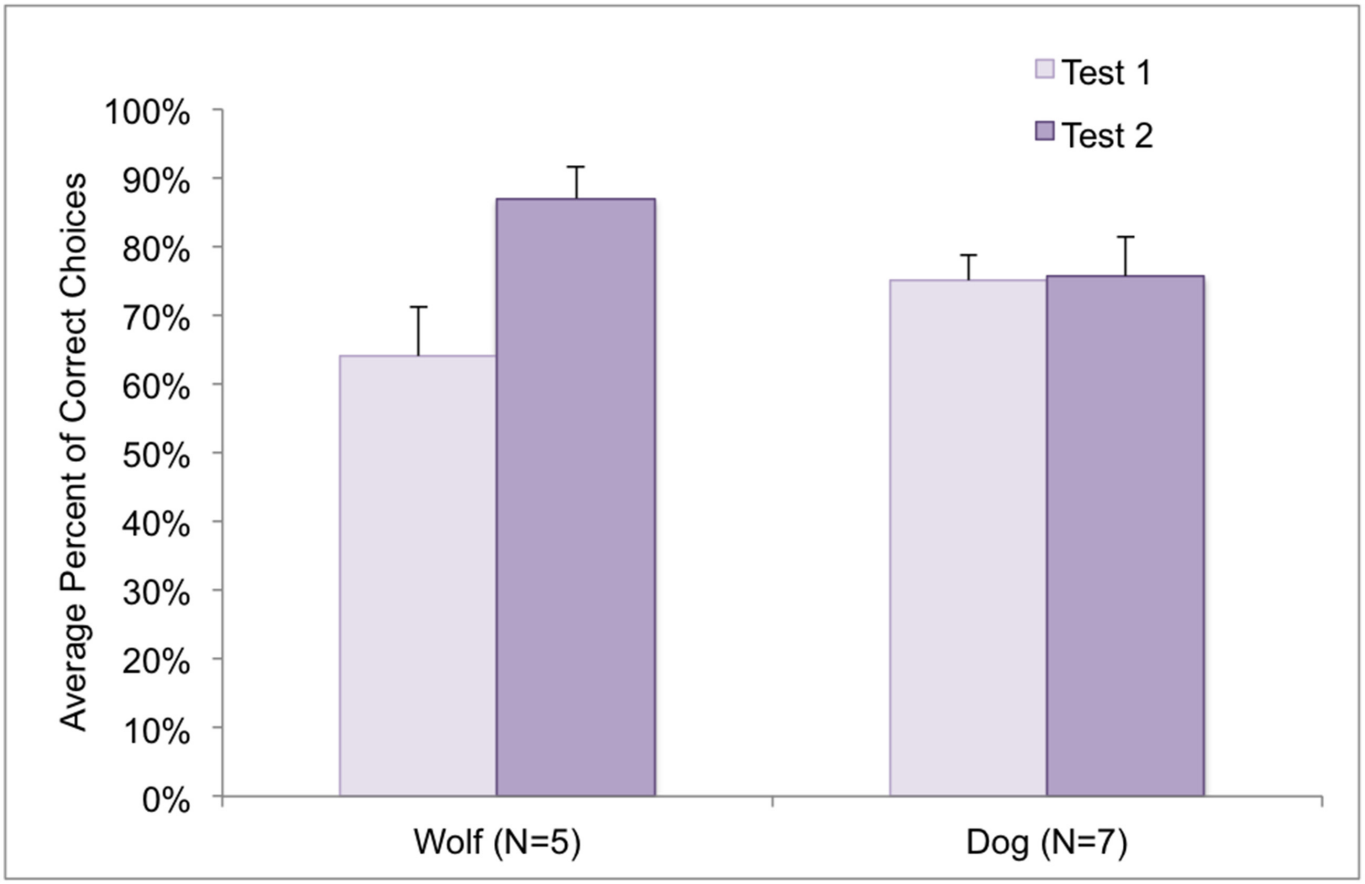
Mean (SE) success rates for the retested animals in Test 1 and Test 2.

Comparing the overall success (average score of all levels) in the first and second testing occasion in case of a mixed sample of dogs (N = 7) and wolves (N = 5) we found difference only in the performances of the wolves (repeat: F_(1,10)_ = 6.557, p = 0.028; repeat x species interaction: F_(1,10)_ = 5.791, p = 0.037) (Fig 4).

**Fig 5:**
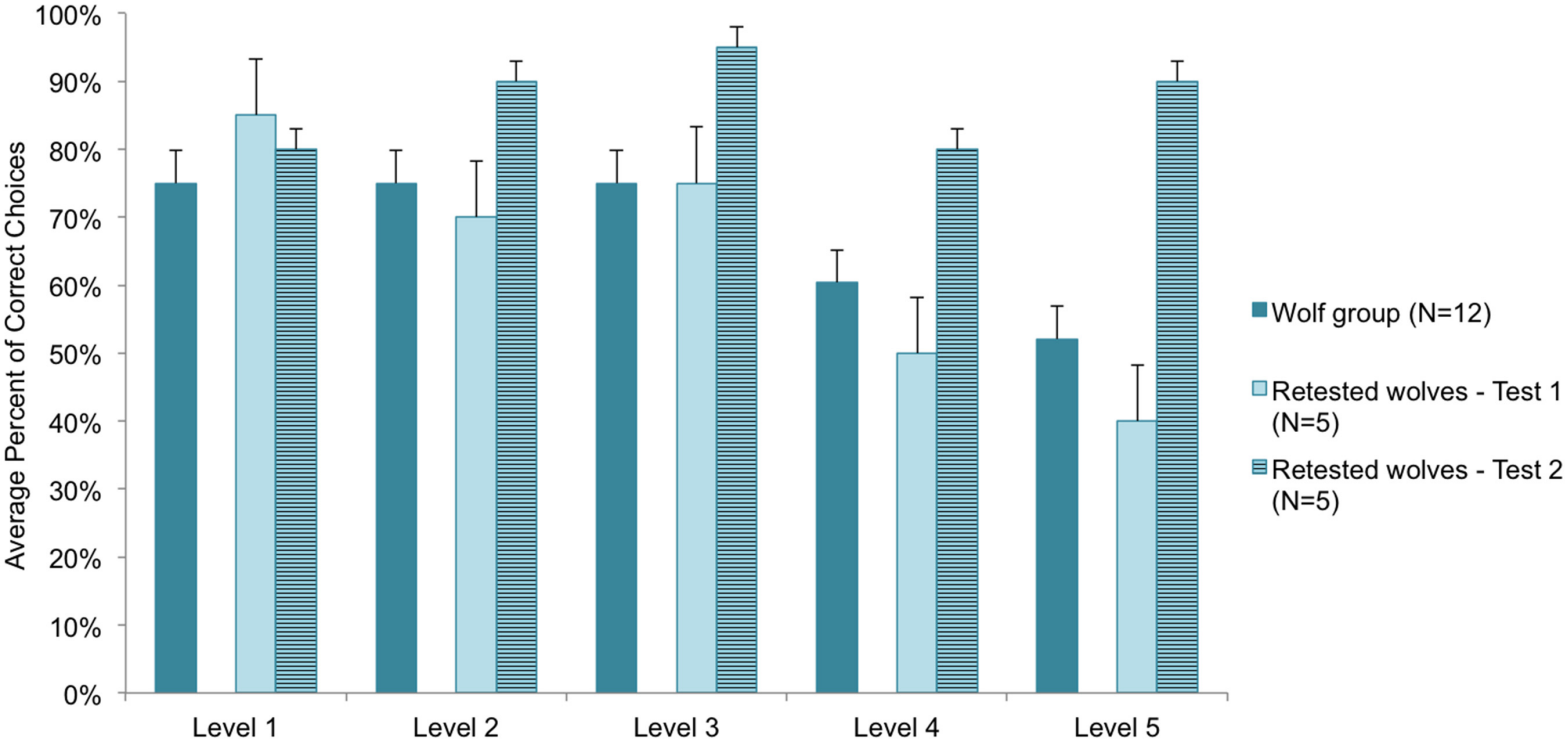
Mean (SE) performance of wolves in the Natural Detection Task. In addition to the average performance of the wolf group as a whole, the performance of the retested animals in Test 1 and Test 2 is shown across the five levels.

Further examination of the performance of the retested wolves (Fig 5) revealed that the increase of their success was more pronounced in the higher levels (level x retest interaction: F_(4,16)_ = 3.571, p = 0.029.

## Discussion

The fact that we could successfully test the olfactory abilities of 85% of companion dogs from many different breeds and 92% of hand raised wolves in a simple procedure without any pre-training is evidence that the Natural Detection Task has the potential to be a practical method for comparing the relative olfactory abilities of different breeds or individuals.

Furthermore, being able to run the test successfully on wolves provided vital information not only about the comparisons between them and domestic dogs, but also about the applicability of our test procedure. The performance of their first tests proved to be surprisingly low, showing no difference from any of the dog groups. However, when a subsample of the wolves was retested, they showed a marked improvement, performing at 90% even in the hardest level. This suggests that for wolves some learning or habituation to the testing environment may be needed before our test is a reliable method of assessing their relative olfactory capabilities. It is unknown whether the wolves performed this way on account of their genetic differences or on account of their different living conditions. While being on leash and interacting with people were regular experiences both for the dogs and these wolves, it might have been more demanding for a wolves to focus on the task in an environment where other olfactory and visual cues could also attract their attention. Previous studies comparing dogs and hand-reared wolves have found that in some situations wolves interacted differently with humans in task situations [28–31].

The retested companion dogs in our study did not improve their performance in Test 2. Although, due to our small retest sample size, it cannot be excluded that there may be breed differences in this respect, on average the dogs’ performance was consistent in test and retest. This suggests that the Natural Detection Task seems to be a relatively reliable measure of pet dog olfactory ability after even just one test, however, future studies should assess the existence of breed differences in greater detail and with larger retest sample sizes to validate this reliability.

The results of our study show that breeds that had been originally specifically selected for scent work do in fact demonstrate a higher olfactory acuity than breeds that had not been selected for such work. Not only were they more successful than the other groups, but they were also the only dog group to perform above chance at the most difficult level. These results suggest that, although modern day dog ownership tends to focus more on suitability for companionship or morphological characteristics, rather than the original function for which the breed was selected [32], scenting breeds have nevertheless retained the abilities for which they were originally bred. This view contrasts with previous research which found no relationship between present-day behavioral breed characteristics and those behavioral characteristics that correspond to the breeds' original functions [33]. However, it does support other research which looked at breed differences with regards to abilities in certain tasks (such as responding to human pointing), rather than temperament or personality [34–36]. This could possibly indicate that physical traits and abilities related to breed function are more stable within breeds than those personality traits that may be related to the same function, however further research is needed to confirm this notion. Our findings also support the assumptions that breeding for short noses has had a detrimental effect on olfaction; causing short-nosed breeds to have the worst performances out of the breed groups we measured. This may be connected to brachycephalia, a condition that generally comes hand-in-hand with having a short-nose, and which is known to have a negative impact on the movement of air through the nasal passages [37,38]. Considering that the ability to gather olfactory information is an important aspect of being a dog, whether or not this apparent reduced ability has any implications for the welfare of these breeds should be further assessed.

Our results regarding the olfactory abilities of short-nosed breeds contrast with those from a recent study comparing the olfactory performances of pugs, German shepherds, and greyhounds [39]. This study found that the pugs significantly outperformed German shepherds in acquiring an odor discrimination task and in maintaining their performance when the concentration of the target odor was reduced. While German shepherds were not included in the present study due to the ambiguity of which of our breed groups they would fall into—‘non-scent’ based on their original function as shepherding dogs, or ‘scent’ based on their current frequent use as detection dogs—neither of these groups were outperformed by the short-nosed group. This discrepancy between the results of these two studies is possibly due to the nature of the tasks that were being assessed. While the Natural Detection Task requires no training and relies on the animal’s inherent motivation to find the target (which is in itself the reward), Hall et al.’s study required extensive training across multiple days to train the dogs to signal for a biologically irrelevant odor (mineral oil) and then receive a reward. This suggests that performance on such an odor discrimination task is dependent not only on olfactory ability, but also on trainability and motivation. This idea is further supported by the fact that 90% of the greyhounds in their study had to be excluded due to their failure to participate, while there was no such trend with the greyhounds or even the non-scent group as a whole in the Natural Detection Task.

Although our results support our hypotheses, we cannot exclude that certain factors apart from olfactory ability may have had an effect on performance. Factors such as fatigue could have decreased the degrees of success in the higher levels or, conversely, experience with the test situation could have enhanced success in later trials. Another possible confounding factor is that some dogs may have been satiated earlier than other dogs—this could be especially true for dogs of smaller sizes. We cannot exclude that there may be differences between breeds and species in which of these factors has an effect. Admittedly, as this study relies on the subjects being motivated to search for the target, it is impossible to perfectly separate the measure of scenting ability from the measure of motivation to participate. We can only assume that any subject who chooses to repeatedly examine the options and eat the bait is in fact motivated to find the target, and that using a direct reward (the searched for target) minimized such an effect.

We believe that the foundations of the procedure we have developed in this study will prove to be a useful tool in future research of canine olfaction. Based on our experiences however, we suggest the following adjustments. First, raw meat was used in our study only because it was the most motivating for the wolves. In studies focusing exclusively on dogs, the use of different food bait, such as some standard type of food could be more practical. Some studies on the olfactory abilities of rats, for example, have used standardized pet food pellets or even commercially available standard cookies or cereals like ‘Fruit Loops’ (Kellogg Company) or ‘Teddy Grahams’ (Nabisco) [40,41]. Similar products could be used for dogs, however it is important that whatever bait is used, it can be standardized across trials to have the same amount of scent and that the scent can be reduced across the levels reliably while still being motivating. There has been some success in using urine scent samples from other dogs as an intrinsically motivating scent [42], however using this scent in the present setup would likely require a new set of indication criteria based on the amount of time spent examining the scent rather than on attempts to obtain the bait. Naturally, the use of any new food baits or scents would require pilot testing with this procedure to confirm its reliability and the optimal quantity to be used.

Being able to run the tests indoors in a controlled environment would also further improve the validity of our methods. This would allow for the further elimination of any distracting scents as well as greater control over temperature, humidity and airflow, all of which may affect the dispersion of scents and may impact measures of olfaction. Indeed, a number of strategies for removing residual odors, such as sterilizing containers and blasting away remaining scents with compressed air, have been developed through previous olfactory studies on dogs and other animals [43]. Moving the test indoors, however, would exclude the further study of most wolves and may prove to be more distracting for some dogs that are kept outdoors and are not used to the various unfamiliar indoor stimuli or confinement (e.g. active hunting dogs).

Additionally, having 20 trials may be quite tiring for some of the animals or some dogs may have been satiated earlier than others. Since we found no significant difference between the performances of the dogs (in any of the breed groups) between the first three levels, including only one of these would likely be sufficient. We suggest using only a three level test: Levels 2, 4, and 5 –that is, the five-holed lid, the one-holed lid, and the closed lid—for a total of 12 trials.

In conclusion, the major advantage of our approach is the speed of testing, allowing for the rapid screening of relative olfactory function in groups of dogs. The Natural Detection Task is a simple and practical method for comparing canine olfactory abilities both on the group level and on the individual level without any pre-training. It has found that breeds of dogs that have been selected for scenting jobs are indeed more successful at this olfactory task, even without any training, than other breeds of dogs that have not been selected for such work. The fact that the dogs did not improve during the retests suggests that the measure is likely reliable for companion dogs after a single test. Future research should improve upon our methods and use them to further advance knowledge of breed and individual differences between dogs in relation to olfactory ability and their potential associations with different genetic and developmental factors.

## Supporting Information

S1 Dataset Data used for statistical analysis. (XLSX)

S1 Video Examples of indications in the wolf sample. (MP4)

S2 Video Examples of indications in the dog sample. (MP4)
